# Improved Short-Term Clock Prediction Method for Real-Time Positioning

**DOI:** 10.3390/s17061308

**Published:** 2017-06-06

**Authors:** Yifei Lv, Zhiqiang Dai, Qile Zhao, Sheng Yang, Jinning Zhou, Jingnan Liu

**Affiliations:** GNSS Research Center, Wuhan University, 129 Luoyu Road, Wuhan 430079, China; lvyifei@whu.edu.cn (Y.L.); iaintyangsheng@gmail.com (S.Y.); zhoujn@whu.edu.cn (J.Z.); jnliu@whu.edu.cn (J.L.)

**Keywords:** real-time precise point positioning, real-time clock estimation, short-term prediction

## Abstract

The application of real-time precise point positioning (PPP) requires real-time precise orbit and clock products that should be predicted within a short time to compensate for the communication delay or data gap. Unlike orbit correction, clock correction is difficult to model and predict. The widely used linear model hardly fits long periodic trends with a small data set and exhibits significant accuracy degradation in real-time prediction when a large data set is used. This study proposes a new prediction model for maintaining short-term satellite clocks to meet the high-precision requirements of real-time clocks and provide clock extrapolation without interrupting the real-time data stream. Fast Fourier transform (FFT) is used to analyze the linear prediction residuals of real-time clocks. The periodic terms obtained through FFT are adopted in the sliding window prediction to achieve a significant improvement in short-term prediction accuracy. This study also analyzes and compares the accuracy of short-term forecasts (less than 3 h) by using different length observations. Experimental results obtained from International GNSS Service (IGS) final products and our own real-time clocks show that the 3-h prediction accuracy is better than 0.85 ns. The new model can replace IGS ultra-rapid products in the application of real-time PPP. It is also found that there is a positive correlation between the prediction accuracy and the short-term stability of on-board clocks. Compared with the accuracy of the traditional linear model, the accuracy of the static PPP using the new model of the 2-h prediction clock in N, E, and U directions is improved by about 50%. Furthermore, the static PPP accuracy of 2-h clock products is better than 0.1 m. When an interruption occurs in the real-time model, the accuracy of the kinematic PPP solution using 1-h clock prediction product is better than 0.2 m, without significant accuracy degradation. This model is of practical significance because it solves the problems of interruption and delay in data broadcast in real-time clock estimation and can meet the requirements of real-time PPP.

## 1. Introduction

Precise point positioning (PPP) has been vigorously developed since its introduction. High-precision orbit and clock products are required by PPP technology [1]. The Global Navigation Satellite System (GNSS) ranging measurement in substance is equal to the time measurement between the satellite clock and receiver clock and usually, the users fix satellite clock corrections and estimate receiver clock corrections to eliminate the error of the clock. In short, the accuracy of the satellite clock is directly related to the accuracy of PPP. 

However, a 12-day to 18-day delay occurs before International GNSS Service (IGS) final orbit and clock products are released, and even IGS rapid products are released after 17 h to 41 h [2,3]. Therefore, such products can only be utilized by users in post PPP applications. Real-time PPP has gradually become a research focus in satellite navigation and positioning, but IGS final and rapid products cannot meet real-time demands. IGS also provides ultra-rapid products (IGU-P). The precision of IGU-predicted orbit products is about 5 cm, which is equivalent to that of IGS final products and satisfies post PPP applications. By contrast, the official accuracy of IGU-P clock products is only 3 ns. Besides, the sampling rate of the clock has a significant impact on the PPP solution in kinematic PPP [4]. Apparently, 15-min interval IGU-P clocks cannot be used for high-precision PPP.

To promote real-time PPP application, IGS set up a real-time working group in 2002 to start the prototype research, and the IGS Real Time Service (RTS) was officially launched in 2013 to provide service for real-time applications [2]. The accuracy of real-time clocks is about 0.3 ns, the broadcast delay is 25 s, and the sampling interval is 5 s [5]. This can be regarded as a reference for real-time clocks estimation. Apart from the integrated products provided by IGS, the analysis center of IGS also provides users their own products. Nevertheless, several problems are encountered in real-time clock applications. First, a delay occurs in real-time observation receiving and product broadcasting. Second, the processing of real-time clock correction estimation software results in a delay. Third, the data stream often loses information or encounters interruption. Table 1 shows the loss rate of real-time clock products in each analysis center between days 349 and 358 in 2015 (except for day 357). The sampling interval is 5 s. In the most ideal case, nearly 10% of the epoch is lost, and this loss seriously affects the accuracy and stability of real-time PPP.

The current Global Positioning System (GPS) constellation can be grouped into three types. Block IIR and Block IIR-M satellites carry rubidium clocks, and the newest generation of Block IIF is equipped with an improving cesium and rubidium atomic frequency standard (AFS). Previous studies have shown that AFS operated on Block IIF is more stable than other types [6,7], and rubidium AFS is more stable than cesium AFS [7,8]. The type of satellite and atomic clock on 1 May 2016 is shown in Table 2. Moreover, the behavior of GPS on-board clocks has been fully discussed by researchers. Periodic signals related to orbital dynamics have been detected in all clock types [9]. Knowing these variations in the satellite clock is important for improving the prediction model of clock.

Most studies and models focused on medium- and long-term forecast analyses, such as one day or several days, the purpose of which is to provide products similar to IGU ultra-fast products to realize real-time PPP with ultra-fast orbit products. This case is obviously different from the short-term clock prediction required for real-time clock difference calculation. Therefore, the introduction of short-term real-time clock correction prediction is important to guarantee the broadcast stability of real-time clock correction and improve real-time PPP accuracy.

Many methods, such as polynomial, gray, autoregressive integrated moving average (ARIMA), and neural network models, can be used for clock error prediction [10,11,12]. The linear or quadratic polynomial model with periodic terms is used as the model of IGU-P products. Huang corrected the original IGU-P clock model, and the accuracy of the improved model is better than that of IGU-P [13]. Several studies focused on short-term real-time clock correction prediction. Lou proposed a polynomial model with periodic items and clock jumps [14]. Song used a polynomial model with periodic items to model the single-difference sequence of clock correction, which was obtained by conducting first-order difference processing on adjacent clock data. The accuracy of this method using 300 s to predict 60 s is better than 0.3 ns [15], which is insufficient for real-time PPP.

This study focuses on short-term clock correction prediction and proposes a new model to improve prediction precision. The prediction residuals between linear sliding forecast and real-time clock product are analyzed and modeled through fast Fourier transform (FFT) [16]. Periodic signals can be eliminated by applying the additional residual model in the subsequent sliding forecast. The experimental results show that with the proposed model, the static PPP achieves precision better than 0.1 m and the kinematic PPP achieves precision better than 0.2 m. This improved method can solve the problems of broadcast delay and interruption in real-time applications.

## 2. Clock Prediction Methods

In this section, we analyze the short-term stability of IGS final clock product, discuss the shortcomings of the traditional model using FFT and propose improved algorithms for short-term clock prediction.

### 2.1. Stability of IGS On-Board Clock Corrections

Although cesium clocks have a better long-term stability than rubidium clocks, the lower short-term noise and price makes the latter more suitable for short-term prediction. The Overlapping Allan DEViation (OADEV), the most common measure of time-domain frequency stability, is used to characterize the stability of three types of GPS on-board clock [17]. In terms of phase data $x_{i}$, the Overlapping Allan VARiance (OAVAR) $\sigma_{y}^{2}$ can be estimated from a set of N time measurements as

(1) $$\sigma_{y}^{2}\left(\tau \right)=\frac{1}{2\left(N-2m\right)\tau^{2}}\overset{N-2m}{\underset{j=1}{\sum }}{\left[x_{i+2m}-2x_{i+m}+x_{i}\right]}^{2}$$

where $\tau \;=\;m\tau_{0}$ is the averaging time interval, m is the averaging factor and $\tau_{0}$ is the basic interval. 

**Figure 1:**
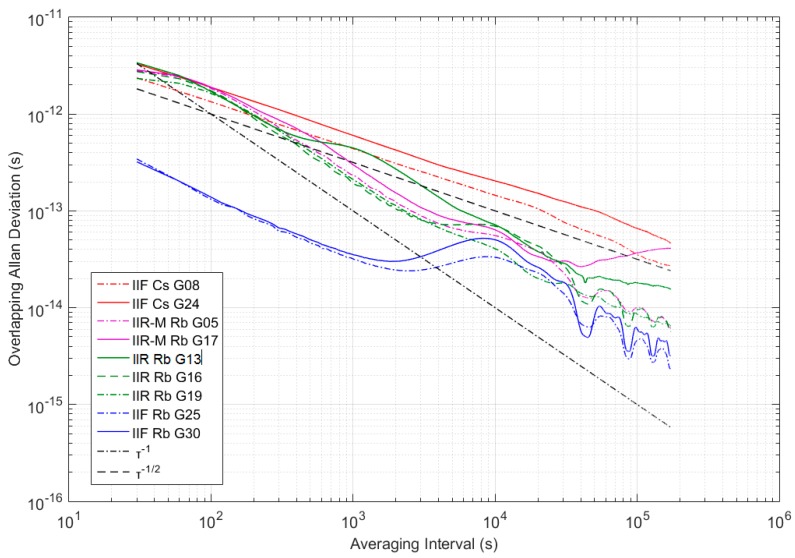
The Overlapping Allan DEViation (OADEV) for GPS on-board clocks over 30 days.

Figure 1 shows OADEV for 30 days IGS clock corrections detrended by removing a second-order polynomial. The stability of the block IIF satellites differs from the previous blocks. The block IIF shows the lowest OADEV of three types of GPS constellation. The OADEV of the block IIF rubidium AFS and cesium AFF varies as $1/\sqrt{\tau }$ up to a few thousand s, consistent with white phase noise. The Block IIR clocks have a very similar performance as the Block IIR-M clocks up to $10^{4}$ s. The decrease of them is closer to 1/τ, which corresponds to white frequency noise. However, it should be noted that G13, launched in 1997, has been shown to differ from others. This may be due to the instability caused by the aging of the atomic clocks. The non-power-law behavior displayed in rubidium AFS at averaging intervals round $10^{4}$ is caused by the 12-h periodic signal [9]. Besides, it can be concluded that the OADEV of GPS constellation varies between $10^{-11}$ and $10^{-14}$ s up to $10^{4}$ s averaging interval. With these short-term features, we can better perform the following analysis of prediction.

### 2.2. Traditional Model

Whether in broadcast ephemeris or practical applications, clock correction is usually modeled by a quadratic polynomial model [18], which can be described as

(2) $$x\left(t\right)\;={\;a}_{0}\;+{\;a}_{1}t\;+{\;a}_{2}t^{2}\;+\;\varepsilon \left(t\right)$$

where x(*t*) is the clock correction at epoch *t;* $a_{0}$ and $a_{1}$ are the clock bias and frequency deviations, respectively; $a_{2}$ is the aging rate or frequency drift; and ε(t) is the generic random noise process. Given that the quadratic polynomial model has a definite physical meaning, it is suitable for clock correction fitting and forecasting in various situations. Considering that short-term prediction requires only a few hours to make the clock relatively stable during this moment, the frequency correction items of the traditional quadratic polynomial model can be disregarded to reduce Equation (2) to

(3) $$x\left(t\right)=a_{0}+a_{1}+\varepsilon \left(t\right)$$

To verify this viewpoint, the root mean square (RMS) of linear and quadratic polynomial models in 5-h short-term prediction is summarized in Table 3. The unit is nanosecond. The time span of validation clock correction is from day 120 to day 151 in 2016, i.e., a total of 30 days of IGS precision clock products. It must be noted that, since the accuracy of the prediction gradually decreases over time, we only evaluate the accuracy of the last forecast epoch. Furthermore, the prediction in this article means sliding prediction, which is used to simulate real-time application. In other words, the 1-h RMS shown in Table 3 is the statistic results of 30 days of 1-h sliding prediction.

**Figure 2:**
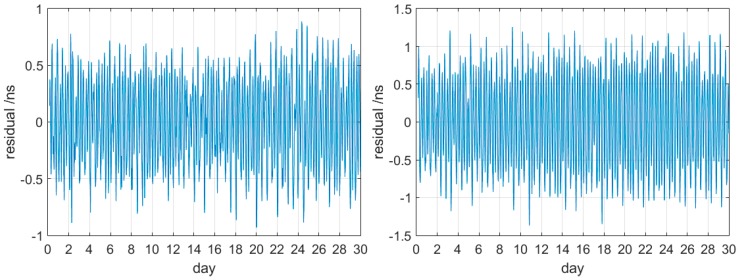
G25 and G30 prediction residuals for 2-h IGS final product.

Table 3 shows that the linear model is more accurate than the quadratic polynomial model regardless of type of satellite and clock. The atomic clock aging does affect the accuracy of G13. The performance of G08 and G24 is much worse than that of the others. Therefore, we discussed short-term prediction of the rubidium clock with the linear model. Considering that the newest Block IIF has better characteristics and would gradually replace other families of satellites, we regarded G25 and G30 as examples of Block IIF to analyze the performance of 2-h linear sliding prediction using 2-h IGS final products. The statistics of 2-h prediction residuals for 30 days are provided in Figure 2.

**Figure 3:**
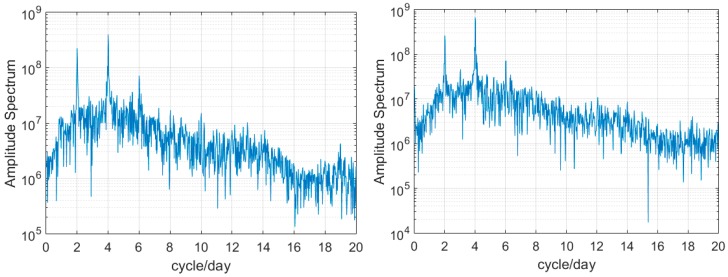
Spectrum of two 2-h prediction residual sequences for G25 and G30.

These significant periodic trends of prediction residuals Figure 2 are also found in other GPS satellites. The use of 2-h clock correction to linearly predict 2-h clock correction can lead to low-frequency and long-term signal effects usually found in continuous forecast. Senior et al. analyzed IGS final clock products with an amplitude spectrum and obtained obvious periodic signals in clock products detrended by removing a second-order polynomial [9]. Therefore, the statistical results of the spectral analysis corresponding to Figure 2 were obtained with FFT and are shown in Figure 3.

Figure 3 shows pronounced 12-h and 6-h peaks along with minimal 4-h variations. The signal variations are similar to the discovery of Senior et al. [9]. The difference is that the current research focused on the linear prediction residuals, whereas the previous research focused on quadratic polynomial fitting residuals. In fact, regardless of the time span of prediction, the corresponding residual cycles are the same as Figure 1. The difference is that the residuals increase with the extrapolation length of prediction. These special characteristics, which involve much time, can damage the accuracy of short-term prediction; however, few-hour data cannot remove long-term variations. Therefore, we propose a new improved model for short-term prediction.

### 2.3. New Model for Short-Term Prediction

If periodic fluctuations in satellite clock sequences are considered, then periodic corrections should be added to the model. In real-time clock prediction, we assume that the clock has similar stability and variations in a short period. The long-term residual sequence of linear prediction is modeled to solve the problem that the few-hour available clock correction is insufficient to obtain low-frequency variations. The coefficients of the periodic term are obtained by fitting the previous long-term prediction results using the results of FFT analysis. The coefficients are then applied to the subsequent forecasting process to improve forecast precision. Equation (3) can be changed to the following formula.

(4) $$x\left(t\right)=a_{0}+a_{1}t+\sum_{i=1}^{N}b_{t,i}\sin\left(\frac{2\pi }{T_{i}}t-t_{0}\right)+\;\sum_{i=1}^{N}c_{t,i}\cos\left(\frac{2\pi }{T_{i}}t-t_{0}\right)+\varepsilon \left(t\right),$$

where $b_{t,i}$ and $c_{t,i}$ (*i* = 1, 2, …, N) are coefficients of the periodic term corresponding to the extrapolated time *t*, which can be obtained by fitting the residual sequence of the previous period with least squares. $t_{0}$ is the initial time of the long-term residual sequence. $T_{i}$ is the corresponding period, and N is the number of periods. Sliding forecast is used in the practical application.

The user can set the extrapolation time according to the demand to densify the prediction sequence. The advantage of this new strategy is that it is possible to estimate a set of periodic term coefficients ($b_{t,i}$ and $c_{t,i}$) for each extrapolated time interval with higher accuracy. The disadvantage is that the longer the predicted length, the greater the computational complexity. It is more suitable for maintaining short-term prediction of the real-time clock.

## 3. Experiment Results

### 3.1. Accuracy Verification of Short-Term Clock Prediction 

In this section, we use the IGS final product to analyze high-precision real-time clock prediction to demonstrate the improved method, and then compare the accuracy with the IGU-P, showing the advantages of the improved model. At last, the practical effectiveness of this model is verified by the real-time clock product.

#### 3.1.1. Experiment Using IGS Final Product

The use of IGS clock final products with 0.075 ns precision provides PPP results at centimeter-level application [18]. These products refer to the IGS timescale [19]. The accuracy of PPP solutions of a single day in E, N, and U directions is about 5, 3, and 9 mm, respectively [20]. We use IGS precision clock products obtained from the IGS website to improve the prediction method and analyze the high-precision real-time clock prediction [21]. The time span of clock correction is from day 120 to day 151 in 2016, which entails a total of 30 days of IGS precision clock products tabulated at 30-s intervals [22]. Table 3 indicates that the prediction results of cesium AFS are not ideal when the linear or quadratic model is used. Therefore, only rubidium AFS is discussed in the next part of this paper.

**Figure 4:**
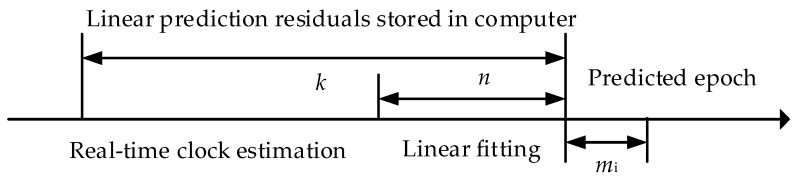
Workflow-diagram for one single epoch $m_{i}$.

During real-time prediction, only a few hours of short-term data are selected to ensure the accuracy of the clock error of adjacent epochs. A prediction workflow-diagram for one single epoch $m_{i}\;\left(i=1,\;2,\;3,\cdots \right)$ is depicted in Figure 4. The processing method is as follows. First, *n* hours clock data are used for the linear sliding prediction of clock correction after $m_{i}$*.* Second, when the prediction is accumulated to *k* hours, the residuals between the prediction and estimation are fitted by the periodic terms obtained through FFT to estimate the coefficients and corresponding time reference. Finally, when the periodic term is added to the subsequent sliding prediction, the prediction accuracy is greatly improved. Figure 3 indicates that the periodic signals of 12 and 6 h are stronger than those of the 4-h variation. Thus, we let the value of N be 2.

**Figure 5:**
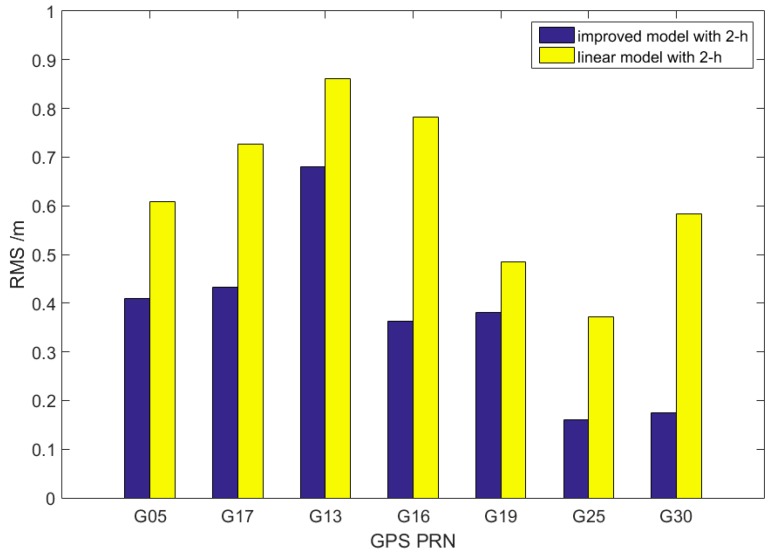
Comparison of 2-h results obtained with improved and linear models.

Figure 5 shows the prediction accuracy of the improved model (blue) and the general linear model (yellow) to intuitively compare the improvement degree. We use 2 h of observation to fit the trend term, forecast the 2-h clock correction, and simulate the real-time clock for 30 days with a continuous 30-day IGS final clock product. The difference is that the improved model is added with the periodic term correction using the residuals of the previous day forecast. All of the satellite clock prediction accuracy depicted in Figure 5 improves. In particular, Block IIF satellites are generally better than 0.3 ns due to their high accuracy. The remaining types of satellites also improve significantly, and several of them, such as G16, exhibit improved accuracy by nearly half. Satellite type does not affect the improvement in model accuracy, and the previous periodic signal model can be applied to the current prediction. 

**Figure 6:**
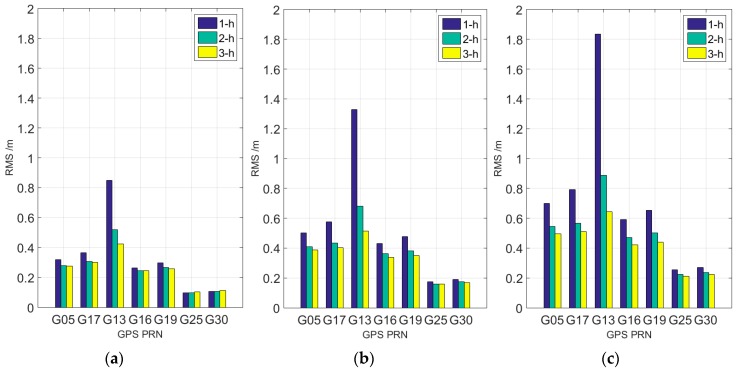
Comparison of 1-h prediction (**a**), 2-h prediction (**b**) and 3-h prediction (**c**) results based on different lengths of observations.

Part of the results of 1-h (blue), 2-h (green), and 3-h (yellow) clock prediction depicted in Figure 6 is used to determine how much observation is suitable for short-term prediction. Figure 6a indicates that precision is concentrated in two ranges. The first range is the rest of the Block IIF satellites. The accuracy of their on-board atomic clocks is better than 0.2 ns, and the length of time required for modeling data exerts a minimal effect on the final results. However, when the linear model is used to forecast 1 h only, the accuracy of using 3-h observation is worse than that of using 1-h observation. In other words, the performance of using 1-h observation shown in Figure 6a is less accurate than that of the two other plans, which demonstrates the effectiveness of the improved model. The second range is the Block IIR and Block IIR-M satellites with precision concentrations between 0.2 and 0.35 ns. Modeling them with 2-h or 3-h observation is better than using the 1-h model, which also confirms that the periodic signal model established with the previous 24-h forecast residuals is suitable for 2-h or 3-h prediction. As for G13, the clock aging should be responsible for the worse accuracy.

The accuracy of the GPS clock prediction for 1-h data modeling in Figure 6b is lower than that of 2-h and 3-h data modeling for two reasons. First, the use of 1-h observation for fitting is insufficient compared with the prediction length. Second, the aforementioned periodic signal model is more suitable for 2-h or 3-h data modeling. Compared with that in Figure 6a, the accuracy of 3-h observation prediction in Figure 6b is generally better than that of 2-h observation prediction. Furthermore, the accuracy of most of the satellite clocks is better than 0.5 ns due to the periodic correction.

The defects and deficiencies of using 1-h observation based on the improved model are obvious in Figure 6c. From the analysis of all the rubidium clocks, except for a few satellites (G28), the accuracy of clock prediction with 3-h data is better than 0.7 ns. The overall accuracy of Block IIF is better than 0.5 ns. It is worth noting that the performance of G13 significantly improved, which can be helpful for predicting the clock aging.

The following conclusions can be drawn from the comparison of Figure 6. First, the newest Block IIF on-board AFS has the best prediction accuracy among Block IIF, Block IIR, and Block IIR-M satellites equipped with rubidium atomic clocks; this result is consistent with the previous studies of short-term stability. Second, the accuracy distribution of each satellite in the three graphs is similar, which may be related to the clock sequence trend. Third, the relative optimal prediction accuracy can be obtained by using 3-h observation modeling, whether it is 1-h, 2-h, or 3-h clock correction forecast. The prediction accuracy of 1, 2, and 3 h is better than 0.45, 0.6, and 0.85 ns, respectively.

#### 3.1.2. Comparison with IGU Product

In practical PPP technology, two approaches can be implemented to acquire real-time clock products. In addition to the real-time clock estimates and forecasts based on the real-time GPS observations, IGU-P products and other similar products can also be used. Therefore, IGU ultra-fast product clocks from day 1 to day 296 in 2016 are sampled to obtain statistics on the 1-h, 2-h, and 3-h clock prediction accuracy of IGU-P products. Forecast precision is evaluated with a method described in literature [23] with reference to IGS post products at a sampling interval of 30 s.

Table 4 shows the improvement of the new model with 3-h observation corresponding to the IGU-P product. The same epoch difference between IGU-P and IGS, identified as outliers, is not analyzed. Table 4 indicates that a decrease in prediction time leads to a significant improvement of the new model. For 3-h prediction, nearly one-third of the satellite forecast results are better than the new model, whereas for 1-h prediction only two satellite results are better than the new model. It is proven that this improved strategy is more suitable for short-term prediction than the IGU-P model. This new strategy can not only reflect the advantages of the linear model in short-term prediction, but also reflect the advantages of the periodic model in long-term prediction. Besides, the type of Block IIF obviously gains more from the improved model, and there is no significant difference between Block IIR and Block IIR-M. In summary, the improved model proposed for real-time clock application can be used to replace IGU-P products.

### 3.2. Experiment Using Real-Time Clock Production

**Figure 7:**
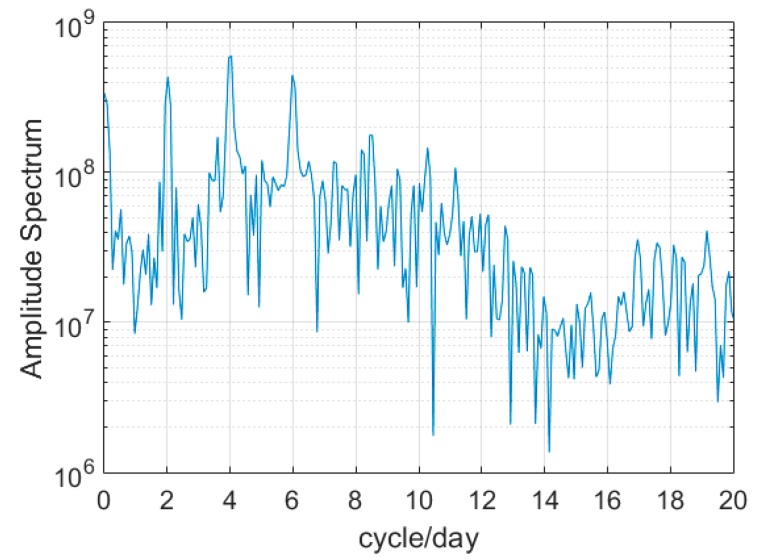
Spectrum analysis for 2-h slipping prediction residuals of G25 after eliminating the clock reference.

The time span of real-time clock corrections is set from day 201 to 206 in 2015 with a sampling interval of 30 s to further verify the availability of the improved model in practical real-time clock application. For the solution strategy, readers can refer to Zhao et al. [23]. The difference from IGS final products is that fitting and prediction using a raw clock correction sequence directly are not ideal due to the impact of the clock basis. To eliminate the reference effect in the prediction, the stable G01 satellite atomic clock is selected as the reference clock to obtain the difference in sequence between G01 and the other satellites. The new improved model is then used to perform a prediction. Figure 7 shows the different signal peaks in real-time linear prediction residuals, and the amplitude spectrum of 4-h prediction is similar to that of 12-h prediction. Therefore, the prediction strategy is changed to extract the long-term trend more effectively, and the 12-h, 6-h, and 4-h periodic terms are selected (*k* = 48 h). *N* and *m* take 1, 2, and 3 h. The prediction results are evaluated with the IGS final clock, in which the clock reference has been eliminated. Based on the analysis of the IGS precise clock, we use the 3-h prediction scheme.

The performance of 1-h, 2-h, and 3-h prediction using the improved model and 3-h observation is shown in Table 5. G08, G10 and G24 carry cesium AFS and G32 belongs to the Block IIA, so all of them are not calculated in Table 5. The accuracy of 1-h, 2-h, and 3-h prediction is less than 0.4, 0.5, and 0.8 ns, respectively. A remarkable finding is that the overall performance of Block IIF remains better than those of other types of satellites.

Reference bias does not reflect clock accuracy. As long as the clock correction of the reference clock does not affect the accuracy of the satellite position, which is better than 1 ms, the system error of the clock can be completely absorbed by receivers in the user position without affecting the final positioning accuracy. The reference bias of the G01 clock can thus be absorbed into the receivers’ clock error and exerts no significant effects on the final positioning accuracy [14]. Therefore, the improved model can be used in the real-time application.

### 3.3. Accuracy Verification of the Static and Kinematic PPP

#### 3.3.1. The Static PPP Tests

An experiment is conducted to evaluate the accuracy of predicted clock correction in the static PPP application. First, a control group using the linear model is selected according to the preceding analysis. Second, the predicted clock correction for 1, 2, and 3 h from day 124 to day 139 in 2016 is calculated and tabulated at 30 s intervals based on IGS final products. Third, five random IGS global distribution-tracking stations (AMC2, CEDU, CHAN, NRC1 and YELL) are selected. Finally, the position and navigation data analysis (PANDA) software developed by Wuhan University is used to process the single-day solution of PPP. For the solution strategy, readers can refer to Ge et al. [24]. The RMS of each station in North (N), East (E), and Up (U) directions is calculated from the station coordinates published on the IGS website. 

**Figure 8:**
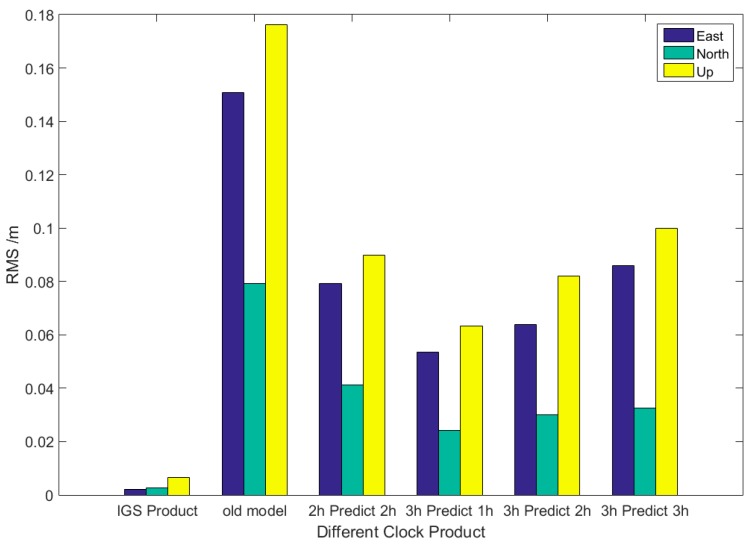
Precise point positioning (PPP) precision of different clock prediction products.

Figure 8 shows the static PPP precision of different clock prediction strategies. The old model is the result of the general linear model for 2-h prediction with 2-h observation. Compared with that based on the IGS final product, the accuracy of the static PPP solution based on forecast products is poorer. The accuracy of the horizontal direction is significantly asymmetric due to the impractical ambiguity resolution of the non-differential single station [9]. Compared with that of the old model, the accuracy of the improved model, the third set of histograms in Figure 8, increases by 47.4%, 47.9%, and 49.1% in N, E, and U directions, respectively. The positioning accuracy of the 2-h linear forecast is lower than that of the 3-h forecast when the improved method is used. The static PPP accuracy of the improved model is better than 0.1 m, which is in sub-decimeter. 

#### 3.3.2. The Kinematic PPP Tests

In order to verify the effectiveness of the improved model in real-time kinematic PPP, two IGS stations (BJFS and CEDU) are used. The raw data is the 24-h observation of day 125, 2016 and the sampling interval is 30 s. The IGS final product of the orbit and clock are used for kinematic PPP solution and clock prediction. We assume that 1-h interruption in the real-time clock caused by an unknown reason occurs at the end of 5 h on day 125, 2016, and the real-time estimated clock is replaced by the prediction clock to realize the real-time kinematic PPP. The residuals of each station in E, N, U directions are calculated from the precision position published on the IGS website.

**Figure 9:**
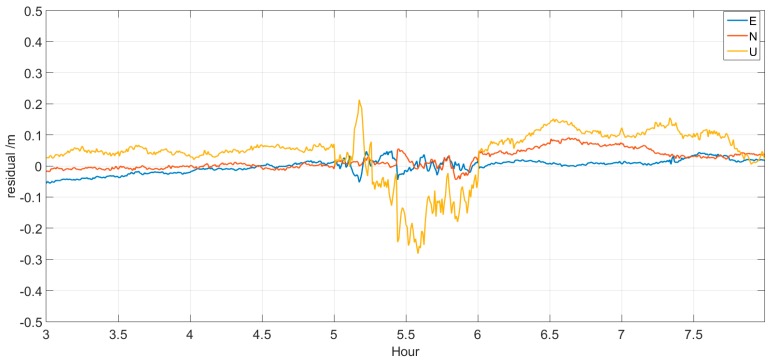
Residuals of kinematic PPP for BJFS.

**Figure 10:**
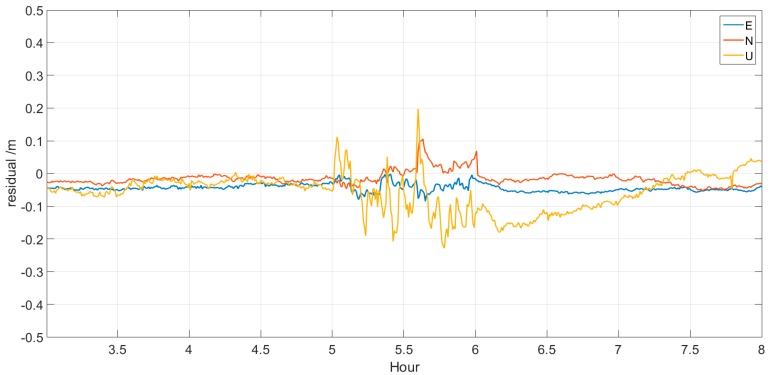
Residuals of kinematic PPP for CEDU.

Figure 9 and Figure 10 show the time series variation of the kinematic PPP residuals of BJFS and CEDU. The accuracy of the first 30 min prediction is obviously better than that of the latter 30 min prediction, and many peaks stand out in the figure. The effect of data interruption on the kinematic PPP solution will continue for some time after the recovery of the real-time clock estimation. However, from Table 6, we can find that the absolute value of residuals in the E, N and U directions is better than 0.3 m, and the std. is better than 0.2 m. It can also be found that the N direction changed the most during the period of prediction. Compared with results of non-prediction, this result proves that the improved model can overcome the problems of interruption and delay in data broadcast in real-time clock estimation and can meet the requirements of real-time PPP.

## 4. Conclusions 

An improved model for short-term prediction based on the traditional linear model is proposed to overcome the problems of data stream interruption and delay in broadcast in real-time clock application. FFT is used to analyze the residuals of the linear prediction and obtain the periodic signals in them. The cumulative prediction residual is modeled, and the periodic term model is applied to the follow-up forecast to improve the prediction accuracy. 

The experimental results using IGS final products show that the 3-h accuracy is better than 0.85 ns. The improved model can thus replace the IGU-P product in real-time PPP. Besides, due to the higher short-term stability, Block IIF exhibits better prediction accuracy than Block IIR and Block IIR-M, and there is no significant difference between Block IIR and Block IIR-M.

Compared with the accuracy of the traditional linear model, the accuracy of the static PPP solution using the new model of the 2-h prediction clock in N, E, and U directions is improved by almost 50%. When an interruption occurs in the real-time model, the accuracy of the kinematic PPP solution using 1-h clock prediction product is better than 0.2 m, without significant accuracy degradation. 

The residuals of linear forecast are modeled to improve the accuracy of subsequent forecasts. According to the experimental results of IGS precision and real-time clock products, we believe that the long-term trend, which is less than the sampling frequency in short-term clock forecast, can also be solved by referring to this method. The disadvantage of this method is that the clock characteristics need to be analyzed carefully before prediction. In general, this new method is simple and accurate and can meet the requirement of real-time clock prediction.

## Figures and Tables

**Table 1:** Loss rate of real-time clock products in each analysis center.

Day	Federal Agency for Cartography and Geodesy (BKG) (%)	Natural Resources Canada (NRCan) (%)	European Space Agency (ESA) (%)	GeoForschungsZentrum (GFZ) Potsdam (%)	Centre National d’Etudes Spatiales (CNES) (%)	IGS (%)
349	43.7	37.9	53.3	44.8	50.8	49.1
350	23.3	23.1	14.2	28.0	35.1	28.3
351	37.5	28.4	30.7	44.4	51.8	33.2
352	10.3	6.7	7.9	6.0	7.5	6.9
353	7.9	4.8	5.5	4.6	5.7	5.5
354	17.3	15.5	16.1	63.4	15.4	15.9
355	13.8	13.2	14.1	47.9	17.0	15.2
356	20.9	12.3	24.2	81.8	21.0	10.5
358	15.6	15.8	17.5	3.3	15.7	15.8

**Table 2:** The type of satellite and atomic clock on 1 May 2016.

Satellite Type	Clock Type	Pseudo Random Noise (PRN)
Block IIR	Rb	2, 11, 13, 14, 16, 18, 19, 20, 21, 22, 23, 28
Block IIR-M	Rb	5, 7, 12, 15, 17, 29, 31
Block IIF	Rb	1, 3, 6, 9, 10, 25, 26, 27, 30, 32
	Cs	8, 24

**Table 3:** Performance of linear and quadratic polynomial models.

		Linear Model (ns)					Quadratic Polynomial Model (ns)				
Type		1 h	2 h	3 h	4 h	5 h	1 h	2 h	3 h	4 h	5 h
IIF Cs	G08	1.226	1.946	2.615	3.316	4.051	2.286	5.375	9.710	15.189	21.982
	G24	1.650	2.647	3.644	4.611	5.562	3.036	7.181	12.948	20.400	29.457
IIR-M Rb	G05	0.351	0.611	0.878	1.113	1.314	0.518	1.228	2.267	3.608	5.220
	G17	0.418	0.725	1.011	1.223	1.362	0.598	1.440	2.673	4.247	6.120
IIR Rb	G13	0.583	0.869	1.180	1.451	1.656	1.475	3.359	6.057	9.540	13.768
	G16	0.417	0.784	1.131	1.405	1.609	0.467	1.209	2.318	3.739	5.395
	G19	0.302	0.486	0.660	0.796	0.893	0.489	1.151	2.095	3.311	4.781
IIF Rb	G25	0.188	0.371	0.548	0.690	0.802	0.190	0.514	1.013	1.648	2.380
	G30	0.291	0.580	0.845	1.028	1.134	0.248	0.728	1.479	2.429	3.495

**Table 4:** Corresponding improvement percentage of the new model.

Improvement Percentage of the New 3-h Model with IGS Product (%)							
**IIF**	**1 h**	**2 h**	**3 h**	**IIR-M**	**1 h**	**2 h**	**3 h**
G01	50.88	39.58	28.66	G05	21.37	15.90	−1.44
G03	63.12	49.27	38.92	G07	17.20	4.84	−0.19
G06	41.06	21.88	0.97	G12	50.55	51.20	50.51
G09	31.38	16.25	1.68	G15	15.73	3.75	−10.59
G10	78.55	72.53	70.03	G17	26.72	24.15	21.38
G25	60.75	50.31	37.20	G19	18.93	9.59	3.29
G26	66.75	60.41	55.23	G31	22.08	16.86	11.87
G27	25.26	25.56	18.30				
G30	58.89	45.83	27.12				
G32	50.29	36.85	37.39				
**IIR**	**1 h**	**2 h**	**3 h**	**IIR**	**1 h**	**2 h**	**3 h**
G02	31.70	27.10	28.11	G19	18.93	9.59	3.29
G11	76.98	75.03	73.52	G20	4.92	−6.74	−20.96
G13	−12.47	−15.77	−19.04	G21	25.42	18.77	−2.99
G14	8.15	−1.42	−8.80	G22	18.84	10.93	2.05
G16	37.18	34.94	16.96	G23	−0.81	−12.78	−24.10
G18	20.58	7.06	−4.09	G28	50.49	46.02	44.36

**Table 5:** The root mean square (RMS) of 1-h, 2-h, and 3-h prediction using the improved model and 3-h observation.

Type	Average (ns)			Max (ns)			Min (ns)		
	1-h	2-h	3-h	1-h	2-h	3-h	1-h	2-h	3-h
Block IIF	0.169	0.248	0.342	0.186	0.273	0.362	0.146	0.226	0.311
Block IIR	0.275	0.376	0.506	0.309	0.464	0.633	0.249	0.334	0.414
Block IIR-M	0.277	0.389	0.525	0.366	0.494	0.709	0.228	0.293	0.391

**Table 6:** Statistics of the kinematic PPP during the prediction period (1-h).

		BJFS			CEDU		
		E (m)	N (m)	U (m)	E (m)	N (m)	U (m)
With prediction	Std	0.022	0.019	0.106	0.020	0.033	0.081
	Max (absolute)	0.053	0.055	0.282	0.085	0.106	0.229
Without prediction	Std	0.009	0.006	0.010	0.005	0.010	0.017
	Max (absolute)	0.041	0.021	0.084	0.036	0.029	0.098
